# The modified functional comorbidity index performed better than the Charlson index and original functional comorbidity index in predicting functional outcome in geriatric rehabilitation: a prospective observational study

**DOI:** 10.1186/s12877-020-1498-z

**Published:** 2020-03-29

**Authors:** Anouk D. Kabboord, Deborah Godfrey, Adam L. Gordon, John R. F. Gladman, Monica Van Eijk, Romke van Balen, Wilco P. Achterberg

**Affiliations:** 1 Department of Public Health and Primary Care, Leiden University Medical Center, Hippocratespad 21, Postbus 9600, 2300 RC Leiden, the Netherlands; 2 Lings Bar Hospital, Nottingham Healthcare NHS Trust, Nottingham, UK; 3 Division of Medical Sciences and Graduate Entry Medicine, University of Nottingham, Nottingham, UK; 4 Nottingham Biomedical Research Centre, University of Nottingham, Nottingham, UK; 5 CLAHRC East Midlands, Nottingham, UK; 6 University Hospitals of Derby and Burton University Hospitals NHS Foundation Trust, Derby, UK; 7 Nottingham University Hospitals NHS Trust, Nottingham, UK; 8 Department of Old-Age Medicine Hubertusduin, HMC Bronovo, The Hague, the Netherlands

## Abstract

**Background:**

In the inpatient rehabilitation of older patients, estimating to what extent the patient may functionally recover (functional prognosis), is important to plan the rehabilitation programme and aid discharge planning. Comorbidity is very common in older patients. However, the role of comorbidity in making a functional prognosis is not clearly defined. The aim of this study was to investigate a modified and weighted Functional Comorbidity Index (w-FCI) in relation to functional recovery and compare its predictive performance with that of the Charlson comorbidity index (CCI) and the original Functional Comorbidity Index (FCI).

**Methods:**

The COOPERATION study (Comorbidity and Outcomes of Older Patients Evaluated in RehabilitATION) is a prospective observational cohort study. Data of patients that were admitted in an inpatient geriatric rehabilitation facility in the UK between January and September 2017, were collected. The outcome measures were: the Elderly Mobility Scale (EMS) and Barthel index (BI) at discharge, EMS gain/day and BI gain/day. Baseline comorbidity was assessed using the CCI, the FCI and the w-FCI. Correlations, receiver operating curves (ROC), and multiple linear regression analyses were performed. The models were adjusted for age, gender and EMS or BI on admission.

**Results:**

In total, 98 patients (mean age 82 years; 37% male) were included. The areas under the ROC curves of the w-FCI (EMS at discharge: 0.72, EMS gain/day: 0.72, BI at discharge: 0.66 and BI gain/day: 0.60) were higher than for the CCI (0.50, 0.53, 0.49, 0.44 respectively) and FCI (0.65, 0.55, 0.60, 0.49 respectively). The w-FCI was independently associated with EMS at discharge (20.7% of variance explained (PVE), *p* < 0.001), EMS gain/day (11.2PVE, *p* < 0.001), and BI at discharge (18.3 PVE, *p* < 0.001). The FCI was only associated with EMS gain/day (3.9 PVE, *p* < 0.05). None of the comorbidity indices contributed significantly to BI gain/day (w-FCI: 2.4 PVE, *p* > 0.05).

**Conclusions:**

The w-FCI was predictive of mobility & function at discharge and mobility gain per day, and outperformed the original FCI and the CCI. The w-FCI could be useful in assessing comorbidity in a personalised way and aid functional prognosis at the start of rehabilitation.

## Background

Making a functional prognosis - estimating to what extent a patient is able to functionally recover - at the start of rehabilitation is important for adequate planning of rehabilitation therapy and timely preparation for discharge. The degree to which recovery can be achieved varies between patients. This is particularly true for older patients after an acute and debilitating illness, for example a hip fracture, sepsis or delirium. Achieving an adequate functional level that enables the patient to perform activities of daily living (ADL), with or without aids and/or home care, is necessary prior to discharge home. Therefore, mobility and functional recovery are important outcomes in the rehabilitation of older people.

Many patient-related factors may contribute to successful or unsuccessful rehabilitation outcomes. These can be medical (multi-morbidity, disease severity), functional (premorbid ADL, baseline function) and social (access to formal care, caregiver availability) [1].

The role of assessing comorbidity in functional prognosis in older patients is not well understood and different co-morbidity indices exist [2–4]. Comorbidity can be expected to contribute to the prediction of functional outcome because it may increase the risk of intercurrent illnesses and therefore impede rehabilitation therapy [4–6]. The Charlson index (CCI) is one of the most widely used comorbidity indices [7, 8]. It includes 19 conditions, each assigned a weight based on their hazard ratio; the total score is the sum of these weighted scores. The index, however, was initially developed to predict mortality and not functional outcome. A number of measures have been designed that may be better associated with functional outcome. Some of these are severity weighted, such as the Cumulative Illness Rating Scale and the Index of Co-Existing Diseases, but they are complex, require specific training, and the use of a comprehensive manual [9, 10]. The Functional Comorbidity index (FCI) has been designed specifically in relation to physical function and is easier and more intuitive to use [11]. It includes 18 diagnoses, counting their presence or absence, resulting in a cumulative sum score: the number of comorbidities. A major limitation is that it does not incorporate a severity weighting which could help improve its accuracy in predicting functional outcome [12]. Furthermore, the index does not include dementia, which is a prevalent condition that influences functional abilities among older patients [13]. To investigate a comorbidity index that is both brief and feasible for use in older patients, a severity-weighted rating scale was added to the original FCI and also dementia was added. As such, this modified and weighted FCI (w-FCI) assesses pre-existent comorbidity (chronic conditions) in combination with its impact on present function.

The present study aims to compare the performance of the w-FCI in an older patient population with that of the original FCI and the CCI in predicting mobility and functional recovery at discharge from geriatric rehabilitation [8].

## Methods

### Setting and design

A prospective observational cohort study was carried out as a service improvement project: COOPERATION, Comorbidity and Outcomes of Older Patients Evaluated in RehabilitATION. The setting was a community hospital-based intermediate care facility that provides inpatient rehabilitation services for older people: Lings Bar Hospital in Nottingham, UK. The multidisciplinary team consisted of an advanced nurse practitioner (ANP), nursing staff, a physician, a speech therapist, a physiotherapist, an occupational therapist, and a social worker.

### Patients

Patients studied were older adults that were referred for inpatient geriatric rehabilitation. No strict age criterion was applied, but all patients had multimorbidity, complex medical problems or were ≥ 65 years old. Formal research consent was not required because this study was conducted as a service improvement project under clinical governance. A sample size of 90 was calculated based upon a minimum sample size of 50 + 8 *k* (where *k* = the number of predictors), including four predictors into a linear regression model and assuming a dropout rate of 10% [14]. Other than the open application of these prognostic indices, which were known to the clinical team, patients received care as usual with no additional intervention.

### Comorbidity assessment

**Fig. 1:**
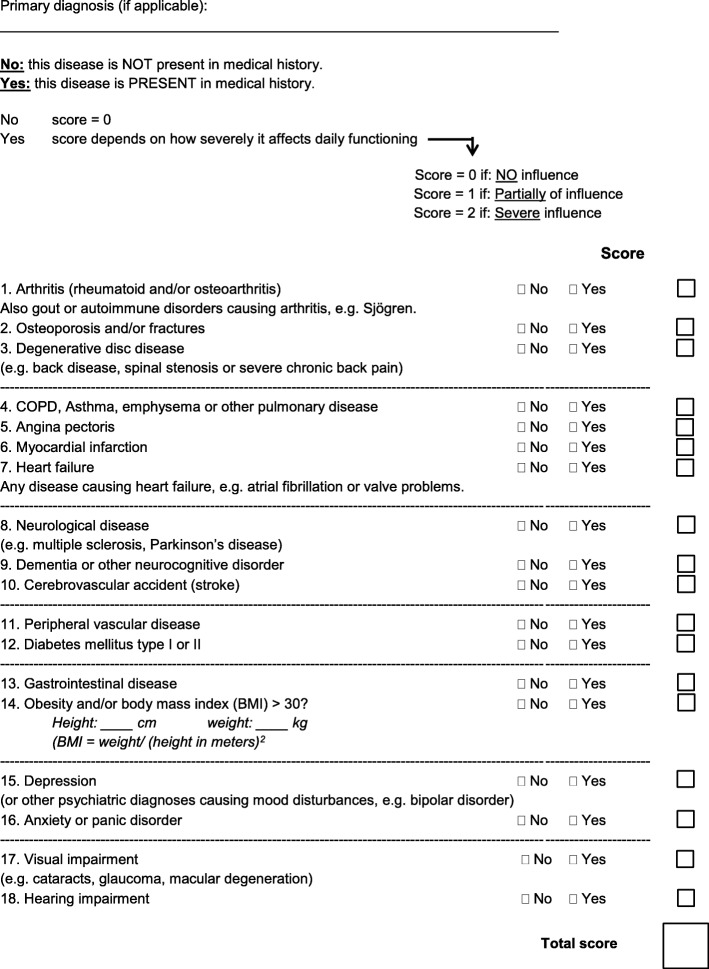
The weighted version of the Functional Comorbidity Index

Pre-existent comorbidity was assessed by the physician or the ANP within the first week of admission using three different indices: the CCI (Additional file 1), the FCI (Additional file 2) and the w-FCI (Fig. 1) [15]. The sum score of the indices represented pre-existent comorbidity and not the actual disease for which the patient had been admitted to the facility.

### Outcome measures

Data from routine clinical assessments were collected on admission and at discharge from the rehabilitation facility. A physiotherapist completed the two outcome measures. These measures from routine clinical data were mobility at discharge as measured with the Elderly Mobility Scale (EMS), range 0–20 (where higher scores denote greater mobility, Additional file 3), from which EMS gain/day was calculated by subtracting EMS on admission from EMS at discharge and dividing the outcome by the total length of stay in days. The other outcome was functional dependency at discharge measured with the Barthel index (BI), range 0–20 (where higher scores denote greater independence in personal ADL, Additional file 4), from which BI gain/day was calculated [16, 17]. The EMS measures mobility and the ability to carry out transfers that are necessary for ADL activities while the patient performs 7 different tasks. The total score depends on the level of help the patient requires to succeed in the tasks. The BI determines the degree of (physical or verbal) help that a person needs to perform ADL activities. Gain/day is a measure that takes account of the fact that the length of stay of each patient varied, leading to a variable time of recovery to which the patient is ‘exposed’.

### Other variables

Besides comorbidity, the ANP collected the following data from routine clinical records in the first week after admission: age, gender, admission domicile, premorbid BI, primary diagnosis, cognition measured using the Montreal Cognitive Assessment (MoCA; range 0–30 where higher scores denote greater cognitive function) [18]. At discharge, the ANP noted the discharge date (length of stay), intercurrent diseases and discharge destination.

### Statistical analysis

The outcomes were used as continuous variables, except for ROC analysis. Correlations (Spearman’s rho) were calculated to test the relation between comorbidity indices and the functional outcome measures. Correlations of 0.1–0.3, 0.3–0.5 and > 0.5 were considered small, medium and large effect sizes respectively [19]. Receiver operating characteristic curve (ROC) analyses were performed in order to create a plot to visualize the differences of predictive performance of the co-morbidity indices. To create the ROC plot, the outcomes were dichotomized: the cut off point for the BI was set at 15 and for the EMS at 13 on the base of literature [20–22]. The clinical interpretation of a BI = 15 is mildly disabled to independent, and EMS = 13 is mildly ADL dependent to independent. For “gain/day” no clinical interpretation of a cut off score is available and therefore was set at their median. Additional ROC curves with different thresholds (cut-off values at 25th, 50th, and 75th percentiles) were created to analyse the robustness of these results. These were performed because AUC’s may vary when different cut-off scores are used. Finally, three multiple linear regression models per outcome were created to compare the R-squared value and percentage of variance explained (PVE) of the w-FCI with that of the other indices. At first, simple models were created (comorbidity index only), age and gender were then added to the second models and function on admission was added to the full models. The areas under the ROC curves (AUCs), R squared values and PVEs were used to compare the performance of the comorbidity indices in relation to the four outcome measures.

## Results

### Characteristics of patients

Ninety-eight patients were included in the study, between January and September 2017. Two patients were admitted directly from home but the remainder was admitted after acute hospitalisation. Fifty-five (56%) patients were admitted following presentation with a fall - with a fracture (*n* = 38) or without fracture (*n* = 17). Patients’ ages ranged from 57 to 99 years and 38 (39%) were male. The median functional level on admission was 5.5 (EMS) and 9 (BI) and this improved to 11 (EMS) and 14 (BI). The median length of stay in the rehabilitation facility was 24 days and functional gain/day was 0.19 (EMS) and 0.18 (BI). In total, 68 (69%) were discharged home. All characteristics are presented in Table 1.

**Table 1:** Patient characteristics on admission and at discharge

On admission (***n*** = 98)	Median (IQR, Q1-Q3) or n (%)
Age	82 (11, 77–88)
Gender (male), n (%)	38 (39)
Admission domicile, n (%)	
- Own home (alone)	40 (41)
- Own home with informal caregiver	31 (32)
- Own home, with formal care assistance	25 (26)
- Other	2 (2)
Premorbid BI	17 (5, 15–20)
CCI	1 (2, 1–3)
Original FCI	3 (2, 2–4)
Weighted FCI	2 (2, 1–3)
MoCA score (baseline)	20 (10, 14–24)
EMS on admission	5.5 (4, 4–8)
BI on admission	9 (5, 6–11)
Primary diagnosis category, n (%)	
- Fall with fracture(s)	38 (39)
- Fall without fracture	17 (17)
- Infectious disease	15 (15)
- Neurological	7 (7)
- Deconditioning	6 (6)
- Other	15 (15)
**At discharge**	
Length of stay (days)	24 (26, 17–43)
EMS at discharge	11 (6, 8–14)
EMS gain/day	0.20 (0.27, 0.11–0.38)
BI at discharge	14 (5, 11–16)
BI gain/day	0.18 (0.22, 0.08–0.30)
Discharge destination, n (%)	
- Own home (alone)	7 (7)
- Own home with informal caregiver	6 (6)
- Own home, with formal care assistance	47 (48)
- Home with health reablement	8 (8)
- Care home	17 (17)
- Transfer to acute hospital (lost to follow up)	3 (3)
- Unknown (missing)	5 (5)
Patients died, n (%)	5 (5)

*Abbreviations: MoCA* Montreal Cognitive Assessment, *EMS* Elderly Mobility Scale

### Comorbidity and functional outcome

The most prevalent comorbidities were arthritis (47%) and osteoporosis (41%). The median scores were 1 for the CCI index (range: 0–8), 3 for the FCI (range: 0–9) and 2 for the w-FCI (range: 0–7). The FCI correlated with both the CCI (ρ: 0.376, *p* < 0.001) and the w-FCI (ρ: 0.497, *p* < 0.001), but the CCI and the w-FCI were not significantly correlated (ρ: 0.180, p: 0.076). The FCI correlated only with EMS at discharge (ρ: − 0.245, p: 0.023). The w-FCI correlated with EMS at discharge (ρ: − 0.469, *p* < 0.001), EMS gain/day (ρ: − 0.385, *p* < 0.001) and BI at discharge (ρ: − 0.415, *p* < 0.001), but did not significantly correlate with BI gain/day (ρ: − 0.125, *p*: 0.250). The CCI did not correlate significantly (*p* > 0.10) with any of the outcomes.

### Predictive performance

**Fig. 2:**
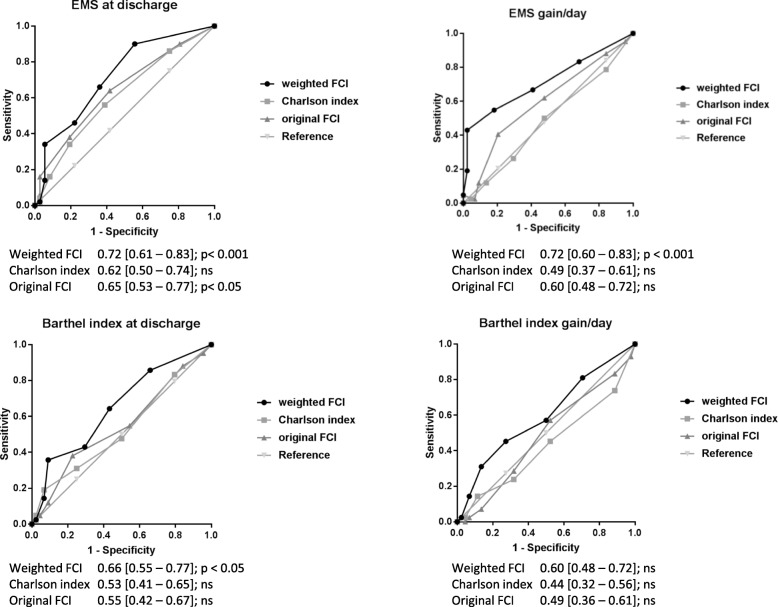
The ROC curves of the four different outcomes

The ROC curves and corresponding AUCs - with their 95% confidence intervals - are presented in Fig. 2.The AUCs of the w-FCI were larger than those of the CCI and the FCI, which applied to all functional outcomes. This remained true for different cut-off scores, except for BI gain/day (Additional file 5). In the linear regression analyses, the CCI did not significantly contribute to the simple or to the full models (*p* > 0.05), the FCI only contributed to EMS gain/day (*p* = 0.037) but was not independently associated in the full models. The w-FCI independently contributed to the prediction of EMS & BI at discharge (*p* < 0.01) and EMS gain/day (*p* < 0.001) but not to BI gain/day (*p* = 0.082). These associations were also statistical significant in the full models. The PVE’s of included variables - with their 95% confidence intervals - are presented in Table 2.

**Table 2:** R squared values and percentages of variance explained per comorbidity index

Simple linear regression	EMS at discharge R^**2**^ (% of variance)	EMS gain/day R^**2**^ (% of variance)	BI at discharge R^**2**^ (% of variance)	BI gain/day R^**2**^ (% of variance)
CCI	0.042 [−0.034–0.118] (4.2%)	0.006 [− 0.024–0.036] (0.6%)	0.037 [− 0.035–0.109] (3.7%)	0.001 [− 0.011–0.013] (0.1%)
Original FCI	0.043 [− 0.034–0.120] (4.3%)	0.051 [− 0.032–0.134] (5.1%)	0.008 [− 0.026–0.042] (0.8%)	0.007 [− 0.025–0.039] (0.7%)
Weighted FCI	0.216 [0.075–0.357] (21.6%)	0.122 [0.003–0.241] (12.2%)	0.192 [0.055–0.329] (19.2%)	0.036 [− 0.035–0.107] (3.6%)
**Multiple linear regression model**				
CCI + age & gender	0.094 [− 0.011–0.199] (9.4%)	0.177 [0.046–0.308] (17.7%)	0.107 [− 0.004–0.218] (10.7%)	0.125 [0.008–0.242] (12.5%)
Original FCI + age & gender	0.082 [− 0.018–0.342] (8.2%)	0.184 [0.051–0.317] (18.4%)	0.080 [− 0.019–0.179] (8.0%)	0.126 [0.008–0.244] (12.6%)
w-FCI + age & gender	0.246 [0.104–0.388] (21.8%)	0.242 [0.101–0.383] (24.3)	0.227 [0.087–0.367] (22.7%)	0.137 [0.016–0.258] (13.7%)
CCI + age, gender & function on admission	0.423 [0.282–0.564] (42.3%)	0.199 [0.065–0.342] (19.9%)	0.460 [0.323–0.597] (46.0%)	0.131 [0.013–0.249] (13.1%)
Original FCI + age, gender & function on admission	0.423 [0.282–0.564] (42.3%)	0.207 [0.072–0.342] (20.7)	0.463 [0.326–0.600] (46.3%)	0.132 [0.014–0.250] (13.2%)
w-FCI + age, gender & function on admission	0.487 [0.353–0.621] (48.7%)	0.291 [0.148–0.434] (29.1%)	0.470 [0.334–0.606] (47.0%)	0.160 [0.034–0.286] (16.0%)

*Abbreviations: EMS* elderly mobility scale, *FCI* functional comorbidity index

## Discussion

### Main findings

Our key finding was that the modified FCI had a better predictive performance than the CCI and the original FCI with regard to EMS and BI at discharge and EMS gain/day in older patients that underwent geriatric rehabilitation. The w-FCI had a larger AUC and stronger correlation with these three outcomes (medium effect size) than the CCI and FCI. Results were not significant for BI gain/day. Furthermore, the w-FCI was independently associated with EMS and BI at discharge and EMS gain/day, whereas the CCI and FCI were not.

### Strengths and limitations

This study has several strengths: we did not apply any restrictions or exclusion criteria except that all patients had to be referred for rehabilitation. The study cohort was characterised by a high age, prevalent comorbidity and a large drop in mobility and functional capacity after acute illness: it was a typical population and the study was conducted in a normal clinical setting [23]. However, no stroke patients were admitted in the facility: stroke rehabilitation usually is provided in specific post-acute stroke rehabilitation facilities.

Furthermore, the design of the w-FCI and its rating scale is function-based and involves the clinical judgement of the clinician. This is in contrast to many studies that used an administration-based method of assessing comorbidity. Therefore, this prospective study gives insight in the clinical assessment of severity-weighted comorbidity and its potency in making a functional prognosis. Finally, we used two different rehabilitation impact indices per outcome measure: function/mobility at discharge and function/mobility gain/day [24]. Function at discharge is an important rehabilitation outcome that indicates the functional independence of a patient, which is necessary for discharge planning. However, other factors than functional status alone may influence discharge planning such as availability of informal caregivers and home situation (stairs or ground floor). That is why EMS and BI gain/day - which is a measure of rehabilitation efficiency - are also important outcomes with regard to the functional prognosis and duration of stay.

There were also several limitations. The study cohort was relatively small and the study was carried out in one facility where the clinicians that completed the measurements were not blinded to clinical practice and the course of a patient. To minimize potential bias, the therapists that performed the EMS and BI were not aware of the comorbidity indices and its scores. Therefore, we think it is unlikely that it has affected our results to any major degree. We also did not take therapy type, duration and intensity into account. Patients likely received customized therapy, on the basis of their capacity and general condition. A larger multicentre study that takes account of therapy differences across patients would be needed to investigate whether the predictive validity of the w-FCI can be confirmed. It is also important to realise that our findings apply to vulnerable older patients but may not be generalizable to younger patients with less comorbidity. One last limitation concerning the study design: our study did not investigate outcomes like quality of life or other indicators of wellbeing, which are also important outcomes of rehabilitation.

Furthermore, a limitation of the w-FCI may relate to what we have stated above as one of its strengths: the w-FCI assesses comorbidity on the base of the clinician’s opinion and quantifies this into a rating scale. This may reduce the reliability and reproducibility due to variability of opinions about the impact of a comorbid condition. Lastly, for the ROC analyses of BI or EMS gain/day we have used the medians as the cut-off. A clinical interpretation of these cut off values is lacking in literature, therefore these results have to be interpreted with caution. However, to give a better insight in all the results from the ROC analyses plots and AUC’s with different thresholds are presented in the additional files (Additional file 5).

### Findings in context

With regard to mobility, the w-FCI showed higher AUCs than the other indices and independently contributed to the prediction of mobility and function at discharge and mobility gain/day. This finding supports the conclusion of other studies that severity of disease should be included in comorbidity assessment [12, 25–29]. The w-FCI contains information on the impact of disease in the patient’s individual situation and therefore quantifies severity of comorbidity: a clinical severity weight. This is in contrast to the method of the design studies of the CCI and FCI [8, 11]. In these studies a statistical weight (relative risk and/or beta coefficient) was used and no clinical severity was added to the index. The statistical weighted count in the original FCI study did not perform much better, but the authors discuss the issue that de FCI does not take the severity of diagnoses into consideration. They agree that severity ratings are likely to provide a better performance, but discuss the practical problems of severity rating. We believe that it is an important part of assessing comorbidity in older patients.

In our study, the w-FCI explained almost half of the variance in three out of the four models (not in BI gain/day). For research purposes in older patients, the w-FCI seems to be preferable compared to the CCI and the FCI when functional outcomes are of interest. The CCI has proven to be a sufficient predictor of mortality and we think that the use of it should be restricted to studies that investigate mortality and survival. The FCI has been designed in relation to function, but has not yet been validated in older patients (e.g. absence of dementia), which may explain the lower predictive performance in our study [13].

Regarding the BI, the w-FCI performed sufficiently (AUC > 0.60) and the other indices were poor. An explanation for the overall stronger associations with mobility (EMS) than with the BI (Table 2) could be that the EMS is sensitive in detecting change (improvement), which was found to be stronger compared to that of the BI [30]. In addition, our study cohort was specifically characterized by reduced mobility (Table 1).

### Interpretation of findings

The present study demonstrated that clinicians were able to estimate functional impact of comorbid conditions in such a way that it proved to be an independent factor in predicting mobility and function at discharge and EMS gain/day. Assessing functionally weighted comorbidity using the rating scale distinguishes the w-FCI from the CCI and the FCI. It resembles usual clinical rehabilitation practice, in which a clinician evaluates disease severity, functional impairments and the potential for successful functional recovery. Using the w-FCI, this could be carried out in a brief and structured way, for example as part of comprehensive geriatric assessment. Finally, the w-FCI seems to fit well into the concept of the International Classification of Functioning, Disability and Health, the ICF framework [1]. This framework defines health by the interactions between conditions, body functions and structures, activities and participation, including environmental and personal factors (Additional file 6).

## Conclusions

The w-FCI had higher predictive performance in relation to functional recovery and efficiency of recovery than the CCI and the original FCI, especially when measured using the EMS. The w-FCI may aid in assessing comorbidity in a personalised way and could be incorporated into routine triaging and discharge planning in the rehabilitation practice of older patients. However, further research is required to investigate whether the predictive validity of the w-FCI can be confirmed.

## Supplementary information

**Additional file 1.** The Charlson comorbidity index: lay out of the Charlson comorbidity index.

**Additional file 2.** The original functional comorbidity index: lay out of the original functional comorbidity index.

**Additional file 3.** The Elderly Mobility Scale: content of the Elderly Mobility Scale.

**Additional file 4.** The Barthel index: content of the Barthel index.

**Additional file 5.** Robustness ofd ROC curves: ROC curves of the four different outcome measures with different thresholds (25th, 50th and 75th percentiles as cut-off scores) to present the robustness of the results.

**Additional file 6.** The ICF framework: the w-FCI embedded in the ICF framework.

## Data Availability

The authors confirm that all relevant data are included in the article or its supplementary information files.

## Abbreviations

ADL: Activities of daily living
ANP: Advanced nurse practitioner
AUC: Area under the curve
BI: Barthel index
CCI: Charlson Comorbidity Index
EMS: Elderly mobility scale
FCI: Functional comorbidity index
ICF: International Classification of Functioning, Disability and Health
MoCA: Montreal Cognitive Assessment
PVE: Percentage of Variance Explained
ROC: Receiver operating characteristic
w-FCI: Weighted functional comorbidity index
