# Supplementary material for: The modified functional comorbidity index performed better than the Charlson index and original functional comorbidity index in predicting functional outcome in geriatric rehabilitation: a prospective observational study
Source: BMC Geriatr. 2020 Mar 29;20:114. doi: 10.1186/s12877-020-1498-z (PMC7104537; doi:10.1186/s12877-020-1498-z)
Supplement: Supplementary file 1 — Additional file 1. The Charlson comorbidity index: lay out of the Charlson comorbidity index. [file 12877_2020_1498_MOESM1_ESM.docx]

**Additional file 1 The Charlson comorbidity index**

| **Condition** | **Score** (weight) |
| --- | --- |
| Myocardial infarction | 1 |
| Congestive heart failure | 1 |
| Peripheral vascular disease | 1 |
| Cerebrovascular disease | 1 |
| Dementia | 1 |
| Chronic pulmonary disease | 1 |
| Connective tissue disease | 1 |
| Ulcer disease | 1 |
| Mild liver disease | 1 |
| Diabetes without end organ damage | 1 |
| Hemiplegia | 2 |
| Moderate or severe renal disease | 2 |
| Diabetes with end organ disease | 2 |
| Any tumor / malignacy | 2 |
| Leukemia | 2 |
| Lymphoma | 2 |
| Moderate or severe liver disease | 3 |
| Metastatic solid tumor | 6 |
| AIDS | 6 |
