# Supplementary material for: The modified functional comorbidity index performed better than the Charlson index and original functional comorbidity index in predicting functional outcome in geriatric rehabilitation: a prospective observational study
Source: BMC Geriatr. 2020 Mar 29;20:114. doi: 10.1186/s12877-020-1498-z (PMC7104537; doi:10.1186/s12877-020-1498-z)
Supplement: Supplementary file 2 — Additional file 2. The original functional comorbidity index: lay out of the original functional comorbidity index. [file 12877_2020_1498_MOESM2_ESM.docx]

**Additional file 2 The original functional comorbidity index**

Please indicate whether a co-morbid condition is present (YES) or absent (NO):

YES: this comorbidity is present

NO: this comorbidity is absent

1. Arthritis (rheumatoid and osteoarthritis) ☐ YES ☐ NO
2. Osteoporosis ☐ YES ☐ NO

--------------------------------------------------------------------------------------------------------------------------

1. Asthma ☐ YES ☐ NO
2. Chronic obstructive pulmonary disease (COPD), acute

respiratory distress syndrome (ARDS), or emphysema ☐ YES ☐ NO

--------------------------------------------------------------------------------------------------------------------------

1. Angina ☐ YES ☐ NO
2. Congestive heart failure (or heart disease) ☐ YES ☐ NO
3. Heart attack (myocardial infarct) ☐ YES ☐ NO

--------------------------------------------------------------------------------------------------------------------------

1. Neurological disease ☐ YES ☐ NO

(such as multiple sclerosis or Parkinson’s)

1. Stroke or transient ischemic attack (TIA) ☐ YES ☐ NO

--------------------------------------------------------------------------------------------------------------------------

1. Peripheral vascular disease ☐ YES ☐ NO
2. Diabetes mellitus types I and II ☐ YES ☐ NO
3. Upper gastrointestinal disease ☐ YES ☐ NO

(ulcer, hernia of the diaphragm, reflux)

--------------------------------------------------------------------------------------------------------------------------

1. Depression ☐ YES ☐ NO
2. Anxiety or panic disorders ☐ YES ☐ NO

--------------------------------------------------------------------------------------------------------------------------

1. Visual impairment ☐ YES ☐ NO

(such as cataracts, glaucoma, macular degeneration)

1. Hearing impairment ☐ YES ☐ NO

(very hard of hearing, even with hearing aids)

--------------------------------------------------------------------------------------------------------------------------

1. Degenerative disc disease ☐ YES ☐ NO

(back disease, spinal stenosis or severe chronic back pain)

1. Obesity and/ or body mass index (BMI) > 30? ☐ YES ☐ NO

Height: ____ m Weight: ____ kg

(BMI = weight/ (height in meters)^2^
