# Supplementary material for: The modified functional comorbidity index performed better than the Charlson index and original functional comorbidity index in predicting functional outcome in geriatric rehabilitation: a prospective observational study
Source: BMC Geriatr. 2020 Mar 29;20:114. doi: 10.1186/s12877-020-1498-z (PMC7104537; doi:10.1186/s12877-020-1498-z)
Supplement: Supplementary file 3 — Additional file 3. The Elderly Mobility Scale: content of the Elderly Mobility Scale. [file 12877_2020_1498_MOESM3_ESM.docx]

**Additional file 3 The Elderly Mobility Scale**

| ELDERLY MOBILITY SCALE | |
| --- | --- |
| **Lying to sitting**  **2** Independent  **1** Needs help of 1 person  **0** Needs help of 2+ people | **Gait**  **3**  Independent (incl. use of sticks)  **2** Independent with frame  **1** Mobile with walking aid but erratic/ unsafe turning  **0** Requires physical assistance or constant supervision |
| **Sitting to lying**  **2** Independent  **1** Needs help of 1 person  **0**  Needs help of 2+ people | **Timed walk**  **3**  Under 15 seconds  **2**  16-30 seconds  **1** over 30 seconds |
| **Sit to stand**  **3**  Independent in under 3 seconds  **2**  Independent in over 3 seconds  **1**  Needs help of 1 person (verbal or physical)  **0** Needs help of 2 + people | **Functional Reach**  **4**  Over 20cm  **2** 10-20cm  **0**  Under 10cm or unable |
| **Standing**  **3** Stands without support & reaches within arm’s length  **2**  Stands without support but needs help to reach  **1** Stands, but requires support  **0** Stands, only with physical support  (1 person) |  |
| **Total score:** | Support = uses upper limbs to steady him / herself. |
