# Supplementary material for: The modified functional comorbidity index performed better than the Charlson index and original functional comorbidity index in predicting functional outcome in geriatric rehabilitation: a prospective observational study
Source: BMC Geriatr. 2020 Mar 29;20:114. doi: 10.1186/s12877-020-1498-z (PMC7104537; doi:10.1186/s12877-020-1498-z)
Supplement: Supplementary file 4 — Additional file 4. The Barthel index: content of the Barthel index. [file 12877_2020_1498_MOESM4_ESM.docx]

**Additional file 4 The Bartel index**

| **Activity** |
| --- |
| **Grooming**  0 = needs help with personal care  1 = independent face/hair/teeth/shaving (implements provided) |
| **Bathing**  0 = dependent  1 = independent (or in shower) |
| **Dressing**  0 = dependent  1 = needs help but can do about half unaided  2 = independent (including buttons, zips, laces, etc.) |
| **Feeding**  0 = unable  1 = needs help cutting spreading butter, etc., or requires modified diet  2 = independent |
| **Toilet use**  0 = dependent  1 = needs some help, but can do something alone  2 = independent (on and off, dressing, wiping) |
| **Bowels**  0 = incontinent (or needs to be given enemas)  1 = occasional accident  2 = continent |
| **Bladder**  0 = incontinent, or catheterized and unable to manage alone  1 = occasional accident (max. once in 24 hour)  2 = continent, or catheterized and manages this alone |
| **Transfers (bed to chair and back)**  0 = unable, no sitting balance  1 = major help (one or two people, physical), can sit  2 = minor help (verbal or physical)  3 = independent |
| **Mobility (on level surfaces)**  0 = immobile or < 50 yards  1 = wheelchair independent, including corners, > 50 yards  2 = walks with help of one person (verbal or physical) > 50 yards  3 = independent (but may use any aid; for example, stick) > 50 yards |
| **Stairs**  0 = unable  1 = needs help (verbal, physical, carrying aid)  2 = independent |
| **Total score** |
