# Supplementary figures and images for: The modified functional comorbidity index performed better than the Charlson index and original functional comorbidity index in predicting functional outcome in geriatric rehabilitation: a prospective observational study
Source: BMC Geriatr. 2020 Mar 29;20:114. doi: 10.1186/s12877-020-1498-z (PMC7104537; doi:10.1186/s12877-020-1498-z)

**Additional file 5 Robustness (different thresholds) of ROC curves**


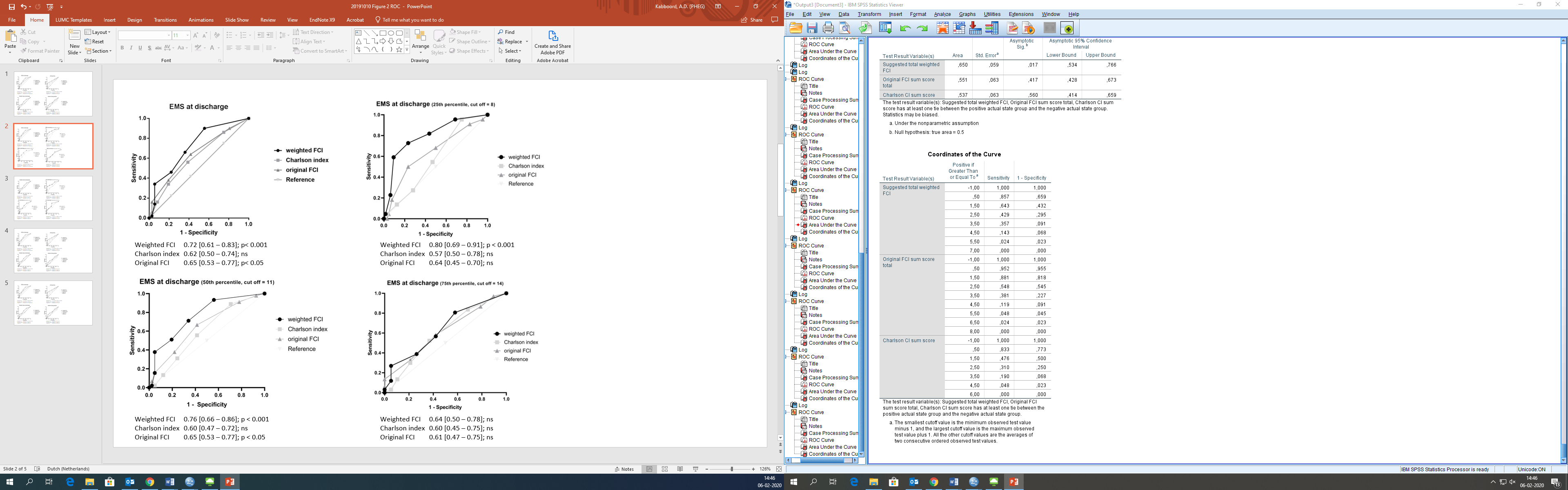


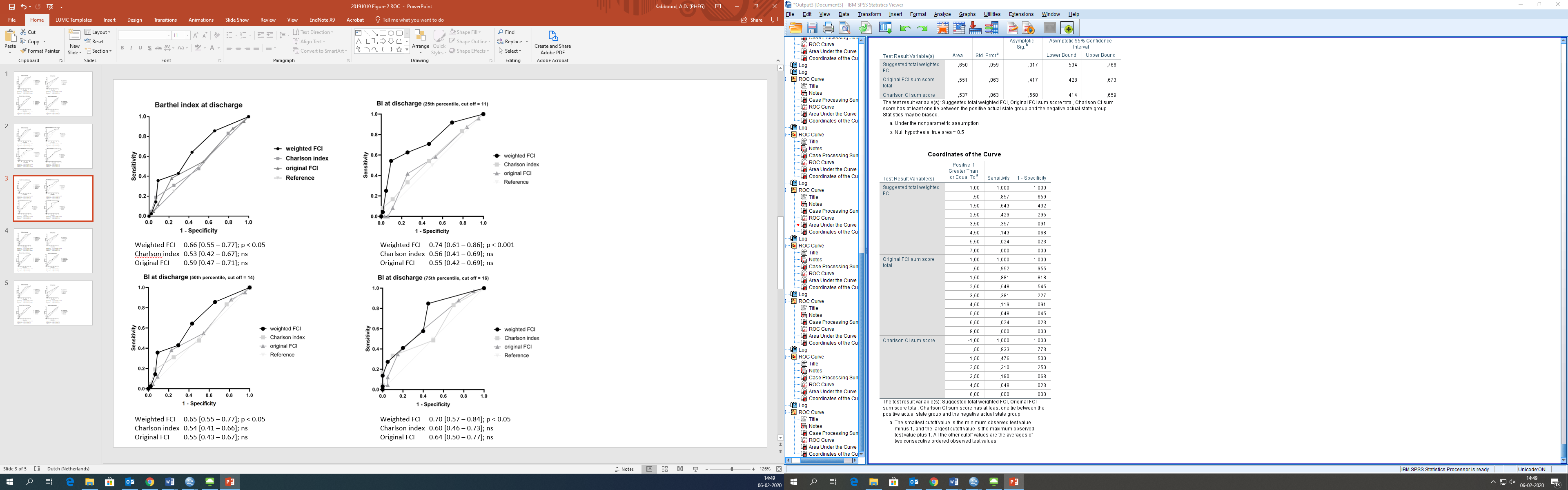


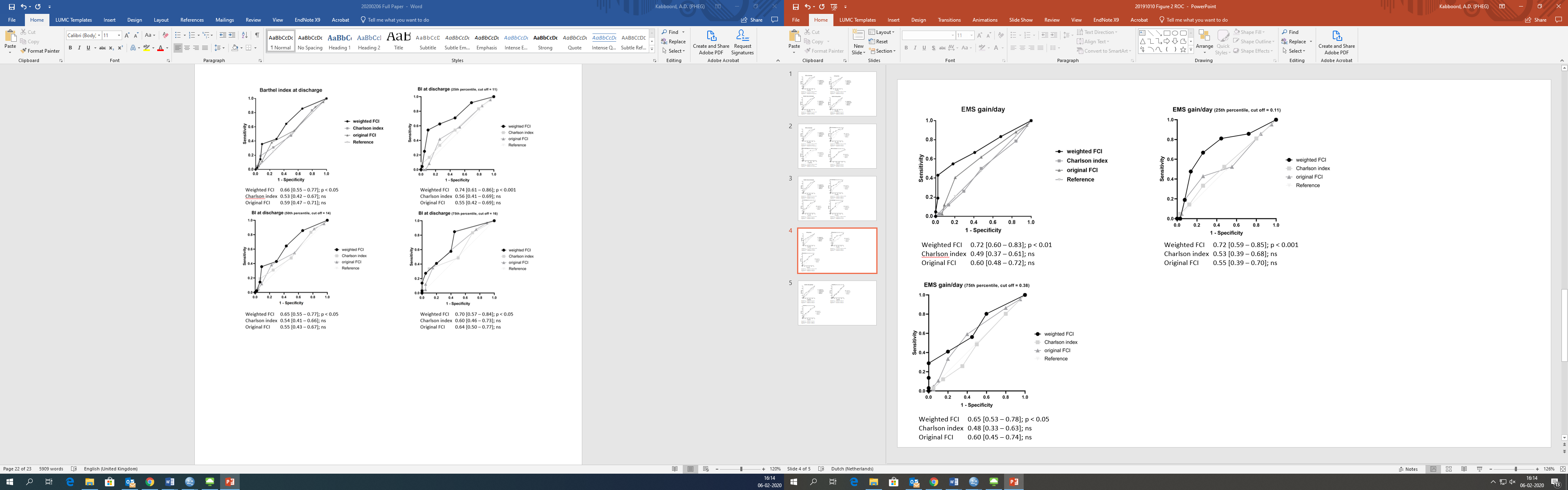


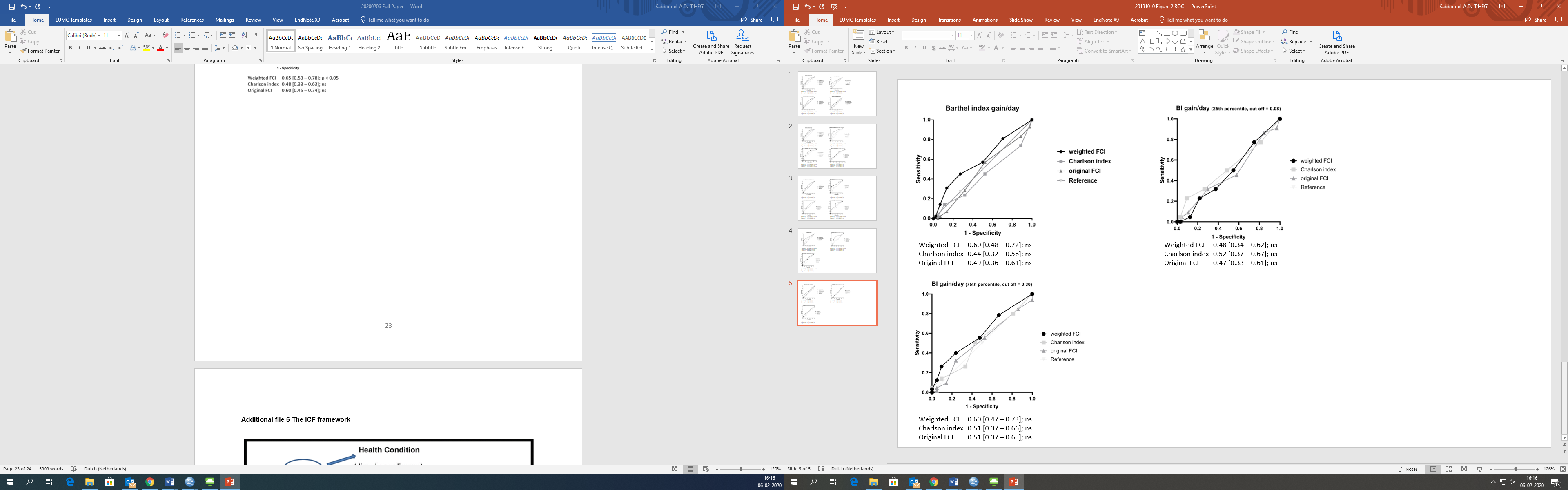

Supplement: Supplementary file 5 — Additional file 5. Robustness ofd ROC curves: ROC curves of the four different outcome measures with different thresholds (25th, 50th and 75th percentiles as cut-off scores) to present the robustness of the results. [file 12877_2020_1498_MOESM5_ESM.docx]

**Additional file 6 The ICF framework**


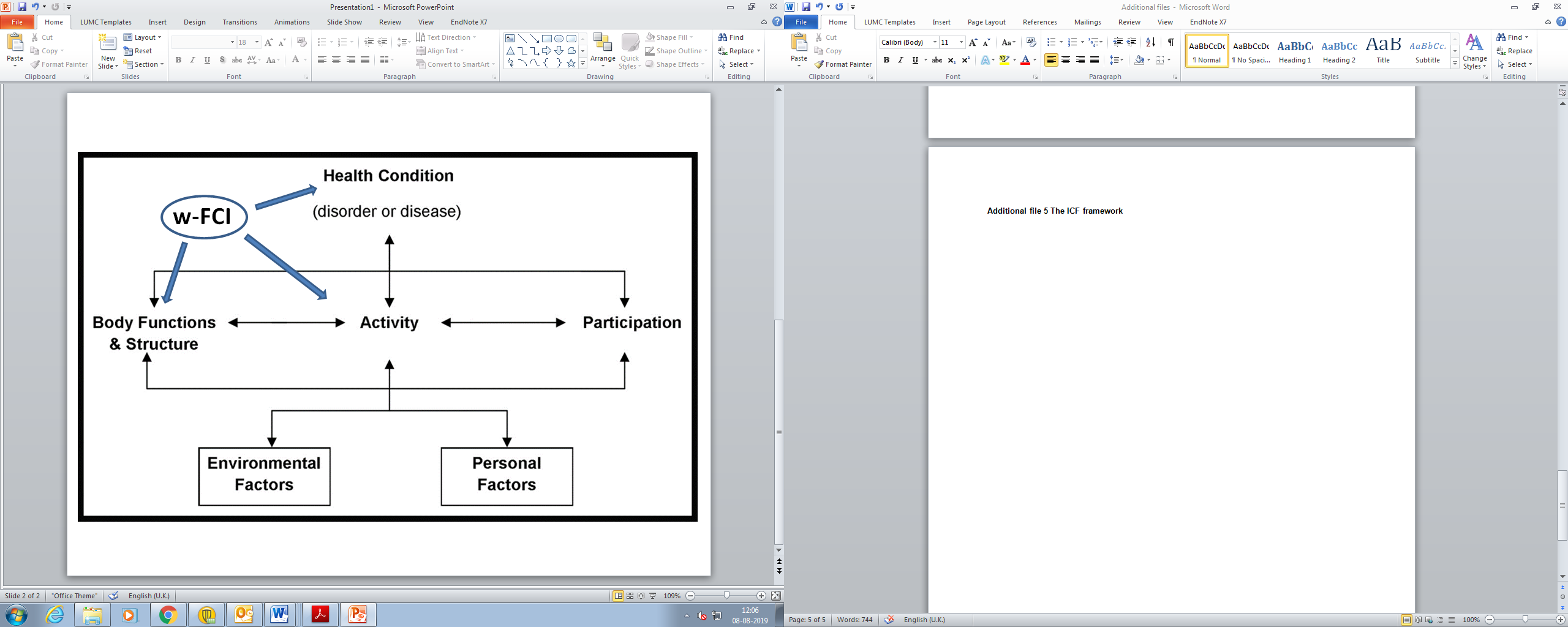

Supplement: Supplementary file 6 — Additional file 6. The ICF framework: the w-FCI embedded in the ICF framework. [file 12877_2020_1498_MOESM6_ESM.docx]
